# Performance and Numerical Simulation of Gel–Foam Systems for Profile Control and Flooding in Fractured Reservoirs

**DOI:** 10.3390/gels12020133

**Published:** 2026-02-02

**Authors:** Junhui Bai, Yingwei He, Jiawei Li, Yue Lang, Zhengxiao Xu, Tongtong Zhang, Qiao Sun, Xun Wei, Fengrui Yang

**Affiliations:** 1 State Key Laboratory of Continental Shale Oil, Daqing 163712, China; baijunhui@petrochina.com.cn (J.B.); hyingwei@petrochina.com.cn (Y.H.); jiaweili@petrochina.com.cn (J.L.); sunqiao01@cnpc.com.cn (Q.S.); weixun@petrochina.com.cn (X.W.); yangfr2009@petrochina.com.cn (F.Y.); 2 Exploration and Development Research Institute of Daqing Oilfield Company Ltd., Daqing 163712, China; 3 Daqing Oilfield Company Ltd., Daqing 163712, China; 4 School of Petroleum and Natural Gas Engineering, Changzhou University, Changzhou 213164, China; ztt55309@163.com

**Keywords:** visualized model, polymer gel, weak cross-linked gel, gravity segregation, enhanced oil recovery, fractured reservoir

## Abstract

Enhanced oil recovery (EOR) in fractured reservoirs presents significant challenges due to fluid channeling and poor sweep efficiency. In this study, a synergistic EOR system was developed with polymer-based weak gel as the primary component and foam as the auxiliary enhancer. The system utilizes a low-concentration polymer (1000 mg·L^−1^) that forms a weakly cross-linked three-dimensional viscoelastic gel network in the aqueous phase, inheriting the core functions of viscosity enhancement and profile control from polymer flooding. Foam acts as an auxiliary component, leveraging the high sweep efficiency and strong displacement capability of gas in fractures. These two components synergistically create a multiscale enhancement mechanism of “bulk-phase stability control and interfacial-driven displacement.” Systematic screening of seven foaming agents identified an optimal formulation of 0.5% SDS and 1000 mg·L^−1^ polymer. Two-dimensional visual flow experiments demonstrated that the polymer-induced gel network significantly improves mobility control and sweep efficiency under various injection volumes (0.1–0.7 PV) and gravity segregation conditions. Numerical simulation in a 3D fractured network model confirmed the superiority of this enhanced system, achieving a final oil recovery rate of 75%, significantly outperforming gas flooding (65%) and water flooding (59%). These findings confirm that weakly cross-linked polymer gels serve as the principal EOR material, with foam providing complementary reinforcement, offering robust conformance control and enhanced recovery potential in fracture-dominated reservoirs.

## 1. Introduction

Enhanced oil recovery (EOR) remains essential for maximizing production from mature and geologically complex fields, where a large fraction of the original oil in place typically persists after primary and secondary recovery stages [1,2]. The challenge is especially acute in naturally fractured reservoirs (notably many carbonates), where most oil resides in low-permeability matrix blocks while injected fluids preferentially channel through high-permeability fractures, causing early breakthrough and poor areal and vertical sweep [3,4]. Conventional water flooding is often ineffective in oil-wet fractured carbonates because spontaneous imbibition of water into the matrix is weak, so the injected water largely bypasses the oil-saturated rock [3,4]. Gas injection (e.g., CO_2_ or hydrocarbon gas) can also suffer from unfavorable mobility ratios, viscous fingering, and gravity segregation, which are problems that are exacerbated in fracture-dominated systems; likewise, water-alternating-gas (WAG) schemes face operational complexity and may still experience override and channeling [2,5]. These limitations highlight the need for improved conformance control and mobility management tailored to fractured reservoir architectures. To address the limitations of water and gas flooding, a more fundamental approach is employed using a polymer-gel dominant system. Here, polymers first enhance water flooding by improving viscosity control. These polymers are then cross-linked into gels to form structural networks for deep conformance control. Foam, serving an auxiliary role, ultimately optimizes gas displacement within this preconditioned reservoir.

Foam flooding has emerged as a promising response to these shortcomings. By dispersing gas as bubbles separated by thin liquid lamellae, a surfactant-stabilized foam greatly increases the apparent viscosity of the gas phase and reduces its mobility, thereby suppressing channeling and gravity override and promoting a more piston-like displacement front [5,6]. Both laboratory and field-scale studies have shown that foams preferentially enter and partially block high-permeability thief zones and fractures, diverting flow into lower-permeability, oil-rich matrix regions and enhancing sweep efficiency [2,5]. At the pore and continuum scales, classical work has quantified the large apparent viscosities attainable with foam in porous structures, providing a mechanistic basis for mobility control and diversion in heterogeneous media [6,7,8]. In fractured carbonates in particular, recent reviews conclude that foam-assisted gas injection can outperform straight gas or WAG by mitigating gas override and improving contact with the matrix [2,5]. Modified surfactant formulations originally designed for foam flooding have shown promise in slow-release pesticide delivery and medicated wound dressings, yet they still await comprehensive safety and cost validation [9,10,11,12]. Recent advancements focus on reinforcing foams through bulk-phase modification. Notably, the integration of polymer gels into the aqueous phase has emerged as a superior strategy. Weak gels can temporarily plug high-permeability channels, enhance localized sweep efficiency, and divert subsequent fluid flow to mobilize oil from other zones, thereby improving liquid absorption performance [13]. The weak gel serves as the main body, inheriting the core functions of polymer viscosity enhancement and profile control from water flooding; the foam acts as an auxiliary component, leveraging the high sweep efficiency and strong displacement capability of gas in fractures. The two synergistically form a multiscale enhancement mechanism of “bulk-phase stability control and interfacial-driven displacement”.

Foam stability is increasingly reinforced through bulk-phase modification using polymer gels, which introduce viscoelasticity, slow drainage, and can even form weak cross-linked networks to stabilize foam films under flow [14,15,16]. Foams are thermodynamically metastable and decay via liquid drainage, bubble coalescence, and gas diffusion (Ostwald ripening). High temperatures, high-salinity brines, and—crucially—contact with crude oil accelerate foam collapse by thinning and rupturing lamellae [17,18]. Xu et al. reported that water-soluble PVA polymers can significantly enhance foamability and stability by promoting surfactant head group hydration and forming a three-dimensional hydrogen bonding network among surfactant molecules, polymer chains, and water [19]. Wu et al. developed a polymer-enhanced foam system based on Z364, a high-temperature-resistant polymer, and CHSB, a zwitterionic surfactant. The system forms a weakly cross-linked network that significantly improves foam stability and mobility control under high-temperature (up to 95 °C) and high-salinity conditions [20]. In large fracture conduits, rapid gravity drainage and segregation can further shorten foam lifetime. Ensuring that foams remain sufficiently stable to propagate, maintain mobility control, and effect diversion over operational timescales is therefore a central challenge for foam EOR, especially in fractured systems [2,21].

Two complementary strategies have gained considerable traction to reinforce foam stability: polymer augmentation of the aqueous phase to enhance viscosity and elasticity, and gas-phase modification to optimize gas composition [22,23,24,25]. Water-soluble polymers increase the viscosity and viscoelasticity of the lamella liquid, slowing drainage and strengthening films; numerous studies have reported improved foam half-life and resistance to oil in polymer-enhanced foams (PEFs), with the caveat that excessive polymer can suppress foamability and alter bubble textures [26,27]. Song et al. engineered a dual-crosslinked polymer gel foam that activates secondary cross-linking under extreme reservoir conditions (140 °C, 24 × 10^4^ mg·L^−1^ salinity), enhancing stability and adaptability [28]. Bai et al. developed an in situ terpolymer/PEI gel system that exhibits thermal stability up to 302 °F (≈150 °C), underscoring the feasibility of forming robust gel-like networks under harsh reservoir conditions [29]. Kamal et al. introduced a novel gel system capable of enduring formation temperatures reaching 150 °C while maintaining stability for approximately 6 h under high shearing rates (511 s^−1^) [30]. Di et al. studied polymer-weak gel oil displacement via NMR flooding, finding that water–polymer-weak gel sequences provided optimal conformance control, increasing oil recovery by up to 18.33% over water flooding baseline [31]. Collectively, this work points toward hybrid formulations—surfactant + polymer—that synergize bulk (viscosity/elasticity) and interfacial stabilization mechanisms to sustain foam performance in reservoir environments [2,32,33].

Building on this foundation, the present study develops a polymer-enhanced foam and evaluates its performance for oil displacement in a fractured network via laboratory testing and numerical simulation. We hypothesize that polymer-based lamella reinforcement will yield a foam that preserves mobility control and diversion in fractures and sustains stability against oil, salinity, and temperature better than conventional foams [34,35,36]. For instance, Pu et al. observed that adding a small amount of polymer to a zwitterionic-surfactant foam sharply increased foam stability at high pressure; in bulk tests with N_2_ at 18 MPa, the polymer-enhanced foam achieved a half-life of ~39 min and exhibited pronounced gains in CO_2_ sensitivity relative to surfactant-only foam [37]. In fractured settings, Telmadarreie and Trivedi further showed that CO_2_ polymer-enhanced foam propagated with higher apparent viscosity and delivered additional heavy-oil recovery and increased CO_2_ storage compared with conventional CO_2_ foam, including in fractured rock samples where diversion into the matrix was evident [38]. These and related findings motivate our polymer concept and its explicit assessment in fracture-dominated flow.

Accordingly, we develop a polymer-based weak gel foam system by incorporating a lightly cross-linked polymer into a surfactant solution, and evaluate its foaming performance, drainage behavior, and oil tolerance under representative conditions. Displacement experiments are conducted in a laboratory fractured-network model to assess the foam’s capacity for mobility control and flow diversion compared to water flooding, gas flooding, and conventional surfactant foam. In parallel, a numerical simulation framework is implemented to investigate foam propagation dynamics, pressure response, and sweep efficiency across fracture–matrix domains. The novelty of this work lies in the synergistic use of polymer-induced viscoelasticity and weak gel structure, which enhances foam stability and enables effective conformance control in fracture-dominated reservoirs. This study thus proposes and validates a polymer gel-stabilized foam formulation, wherein a weakly cross-linked gel network derived from water-soluble polymers enhances foam viscoelasticity, durability, and mobility control in fractured reservoirs.

## 2. Results and Discussion

### 2.1. Formulation of Weak Gel Foam

**Figure 1:**
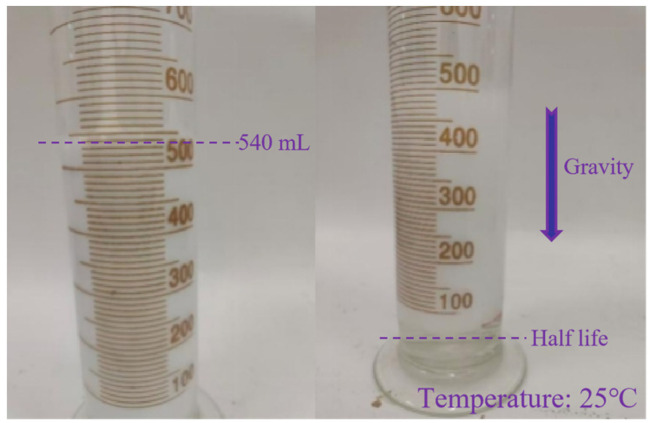
Photograph of the polymer foaming volume and after drainage of 50 mL of liquid.

To enhance the stability of foam for EOR applications, a polymer-induced weak gel network was developed within the lamellae of the foam structure. This gel network was formed through weak cross-linking in the aqueous phase, significantly improving foam drainage resistance, lamella viscoelasticity, and film mechanical strength. As shown in Figure 1, the foam generated from the polymer solution is composed of fine, uniform, and dense bubbles with no apparent large bubbles. The foaming volume is 540 mL. The drainage half-life, which is the time required for 50 mL of liquid to drain, is 32 min and 37 s (equivalent to 1957 s). This results in a Comprehensive Foam Index of 1,056,780 mL·s.

In comparison to the synergistic effect with core–shell particles, the addition of the polymer solution increases the Comprehensive Foam Index from 184,240 mL·s to 1,056,780 mL·s. Based on this improvement, the selected formula is a solution of 0.5% SDS + 1000 mg·L^−1^ polymer.

**Figure 2:**
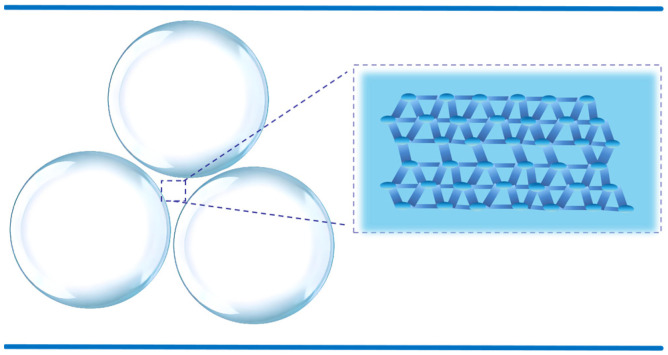
Schematic representation of the gel network structure within the lamella phase.

The foaming volume of the 0.5% SDS solution is greater than that of the solution containing 0.5% SDS plus 1000 mg∙L^−1^ of polymer. The addition of the high-molecular-weight polymer significantly increases the viscosity of the liquid, which slows the diffusion rate of gas and, in turn, decreases foaming efficiency. The slight reduction in foamability upon polymer addition is attributed to increased bulk viscosity and gel structure formation, which dampen gas dispersion during foaming. As shown in Figure 2, the gel network structure within the lamella phase is displayed. This gel network helps to enhance the mechanical strength of the foam and slows down liquid drainage, thereby improving the stability of the foam.

**Figure 3:**
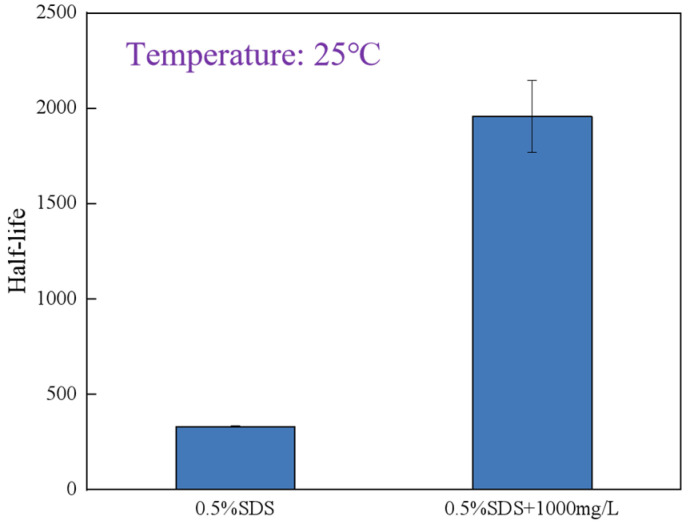
Comparison of half-life between 0.5% SDS and 0.5% SDS + 1000 mg∙L^−1^ polymer solution.

As shown in Figure 3, the half-life of the 0.5% SDS solution is shorter than that of the solution containing 0.5% SDS with 1000 mg∙L^−1^ of polymer. The polymer forms a network structure within the bubble films, which enhances the mechanical strength and viscoelastic properties of the liquid film. This slows the liquid drainage process, thereby increasing the drainage half-life. The weak gel network within the lamella phase enhances mechanical robustness and slows liquid drainage, thereby improving overall foam longevity.

**Figure 4:**
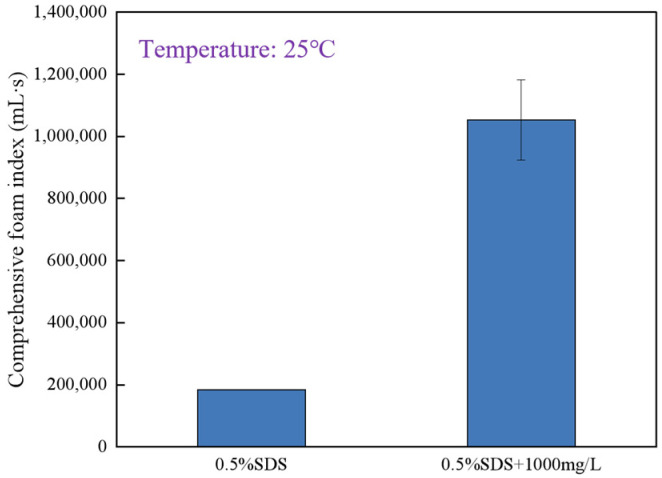
Comparison of the Comprehensive Foam Index between 0.5% SDS and 0.5% SDS + 1000 mg∙L^−1^ polymer solution.

As shown in Figure 4, the Comprehensive Foam Index of the 0.5% SDS solution is lower than that of the solution containing 0.5% SDS with 1000 mg∙L^−1^ of polymer. Therefore, the formula selected for the subsequent flow experiments is a solution of 0.5% SDS and 1000 mg∙L^−1^ polymer.

### 2.2. Foam Flow Experiment Evaluation

The foam flow visualization experiment was performed after water and gas flooding, and the injection rate was kept constant at 2 mL·min^−1^. The experimental design uses both horizontal and vertical two-dimensional models to systematically evaluate the impact of different foam injection volumes and gravity differentiation on fluid migration characteristics and sweep efficiency.

#### 2.2.1. Effects of Different Injection Volumes

**Figure 5:**
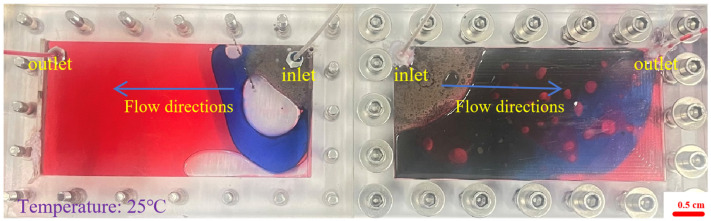
Foam distribution of horizontal model under injection of 0.1 PV foamed gel (same fixed channel/zone geometry; blue phase indicates diversion/sweep expansion; the red phase = the simulated crude oil.): (**Left**): high aperture fractures; (**Right**): small aperture fractures.

**Figure 6:**
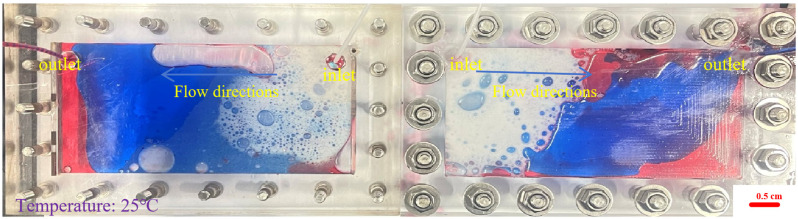
Foam distribution of horizontal model under injection of 0.3 PV polymer foam (fixed high-perm channel + low-perm zone; geometry unchanged, blue phase indicates diversion/sweep expansion; the red phase = the simulated crude oil): (**Left**): high aperture fractures; (**Right**): small aperture fractures.

**Figure 7:**
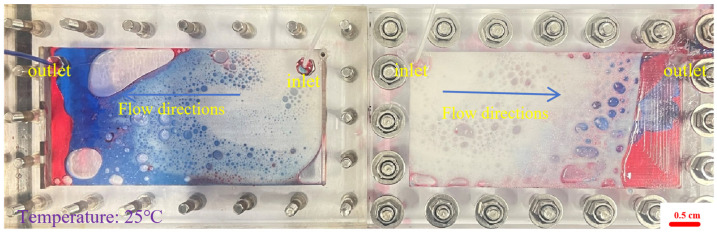
Foam distribution of horizontal model under injection of 0.7 PV polymer foam (more uniform blue phase = diversion/sweep into the predefined low-perm zone; the red phase = the simulated crude oil): (**Left**): high aperture fractures; (**Right**): small aperture fractures.

During the foam flow process, the influence of the injection volume was explored, the visual model was placed horizontally, and the foam was injected at a total speed of 2 mL·min^−1^. The injection volumes of the foam fluid were set to 0.3 PV, 0.7 PV, and 0.1 PV, respectively, and the foam injection model process was obtained for different volumes of foam, as shown in Figure 5, Figure 6 and Figure 7.

From Figure 5, it can be seen that the performance of the 0.1 PV foamed gel is different. The black foamed gel quickly fills the hyperosmotic channel at the beginning of injection, forming a strong seal, forcing the blue water phase to divert the route and the red oil phase to be directly cut off or compressed. The foamed gel achieves efficient sealing under extremely small injection volume, showing a high residual resistance factor, but lacks the surface propulsion advantages of large-volume polymer foam. From Figure 6, it can be seen that when the foam injection amount is 0.3 PV, the white foam preferentially gathers in the hyperosmotic channel to form a local foam plug. The blue water phase is diverted into the low-permeability zone, and the red oil band is sheared into the lenticular residual oil, indicating that the foam has established preliminary fluidity control, but the impact range is limited. As can be seen from Figure 7, as the injection amount increases to 0.7 PV, the foam forms a large-scale dense foam belt in the model, the leading edge tends to piston propulsion, the red oil phase is further dispersed into small oil droplets, and the aqueous phase is diverted and penetrates more deeply and broadly into the predefined low-permeability zone (outside the dominant high-permeability channel), indicating an enlarged sweep due to mobility control rather than any lateral permeability variability. The surface propulsion of foam flooding is significantly enhanced, the horizontal ripple degree is improved, the resistance factor is stable at a high value, and the oil displacement efficiency is significantly improved. Therefore, in practical applications, polymer foam is suitable for medium- to large-volume injection to expand the impact, while foamed gel is more suitable for short-volume rapid “stand-blocking”, and the two combine to form a complementary effect.

#### 2.2.2. Effect of Gravity Differentiation

**Figure 8:**
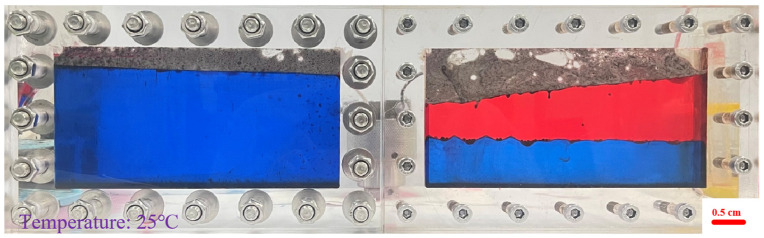
Foam distribution of vertical model under injection of 0.6 PV foamed gel (the red phase = the simulated crude oil): (**Left**): high aperture fractures; (**Right**): small aperture fractures.

At a constant injection rate of 2 mL·min^−1^, foam flow experiments were performed on the visual model in horizontal and vertical placement modes to evaluate the effect of gravity differentiation on flow behavior, as shown in Figure 8.

From Figure 8, it can be seen that the black foamed gel preferentially gathers in the gas channeling path at the upper part of the model to form a dense rubber plug barrier; the upper red oil phase is compressed into strips and gradually broken, the lower blue water phase gradually thickens and advances downstream, and the displacement leading edge tends to flatten. It can be seen that under the action of gravity, the originally obvious gas cap is filled and weakened by foam, the upward channel of gas is continuously suppressed, the vertical sweep degree is improved, and the gas breakthrough is delayed.

### 2.3. Simulation of Oil Displacement Performance

#### 2.3.1. Gas Flooding

**Figure 9:**
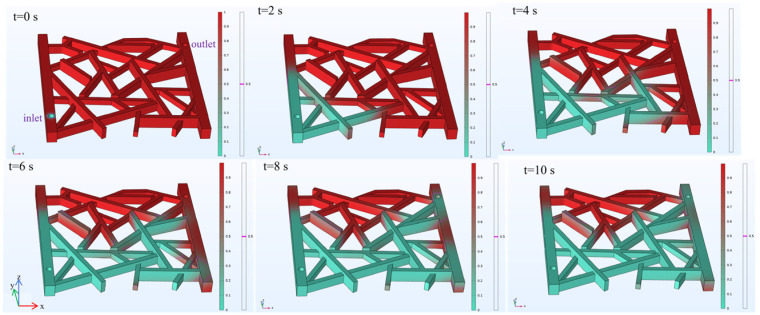
Fluid distribution during the gas flooding process.

The simulation of the gas flooding process models the distribution of the gas (nitrogen) and oil phases. In the visualizations, red represents the simulated oil, and green represents the nitrogen gas. A magenta line indicates the position where the volume fraction between the two fluids is 50%. A boundary probe is added at the model outlet to record the change in gas content during the displacement, while a domain probe is used on the entire model to track the change in the recovery rate. As shown in Figure 9, snapshots of the two-phase fluid distribution are taken every 2 s to observe the dynamic changes over the 10 s period. Due to gravity segregation, the gas phase is distributed above the oil phase in the z-axis direction. It should be noted that this gravity effect mainly manifests as vertical (z-direction) stratification, whereas the visible footprint of phase migration in the current rendering is strongly influenced by the connected fracture-network pathways.

Figure 9 displays a 3D perspective rendering of the fractured-network model for visualization clarity; the model itself is horizontal, not tilted, and the apparent inclination is caused by the viewing angle. Gravity acts along the z-direction, resulting in vertical segregation with gas distributed above oil. Although buoyancy tends to drive gas upward, the imposed inlet pressure gradient promotes pressure-driven flow through connected fracture pathways, allowing gas to invade lower connected branches and displace oil even below the injection point. The scattered residual oil observed at later times mainly arises from local bypassing, limited fracture connectivity, and capillary trapping.

The apparent high efficiency of gas flooding in displacing oil comes from rapid access of gas to the connected fractures under the applied pressure gradient, rather than the absence of gravity effects; gravity still induces upward segregation, but is less visible in the current rendering because the visualization emphasizes horizontal fracture connectivity. The magenta contour denotes the 50% gas–oil volume-fraction interface, which may appear faint due to 3D rendering overlap and color blending. In the revision, the figure caption and text will clarify these details, and, if possible, the magenta interface will be highlighted by thicker lines or an inset view to improve visibility.

**Figure 10:**
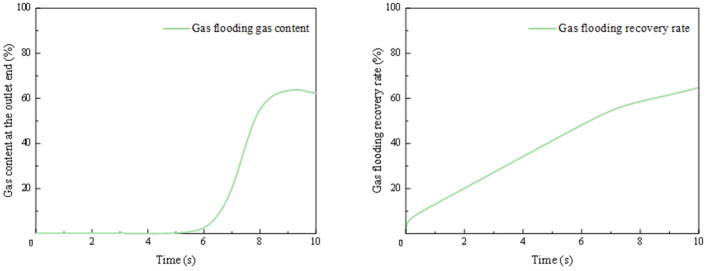
Gas content and recovery rate during gas flooding.

The results are presented in Figure 10. The gas content at the outlet is 0 for the first 5 s. Between 5 and 9 s, the gas content gradually rises to a maximum of 63%. It then decreases slightly to 62% by the 10 s mark. The recovery rate increases rapidly to 6% within the first 0.1 s. It then rises slowly to 56% by the 7.5 s mark. The rate of increase becomes more gradual after that, reaching a final value of 65% at 10 s.

#### 2.3.2. Water Flooding

**Figure 11:**
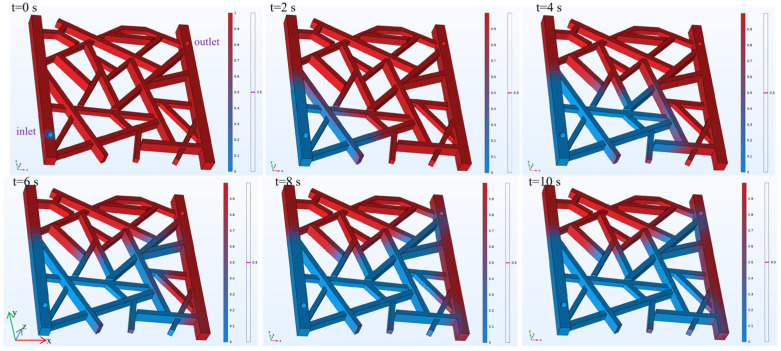
Fluid distribution during the water flooding process.

The simulation of the water flooding process models the distribution of the water and oil phases. In the visualizations, red represents the simulated oil, and blue represents water. A magenta line indicates the 50% volume fraction interface between the two fluids. To monitor the process, a boundary probe is added at the model outlet to record changes in water content, while a domain probe is applied to the entire model to track the oil recovery rate. As shown in Figure 11, snapshots of the fluid distribution are taken every 2 s to observe the dynamic changes over the 10 s simulation. Due to gravity segregation, the water phase is distributed below the oil phase along the z-axis. Although the multiphase distributions in Figure 9 (gas flooding) and Figure 11 (water flooding) may appear similar in the shown snapshots, this does not imply negligible gravity. The apparent similarity mainly arises because the current visualization emphasizes the preferential flow paths controlled by fracture connectivity, while gravity-driven segregation occurs primarily along the z-direction (gas override vs. water underride).

**Figure 12:**
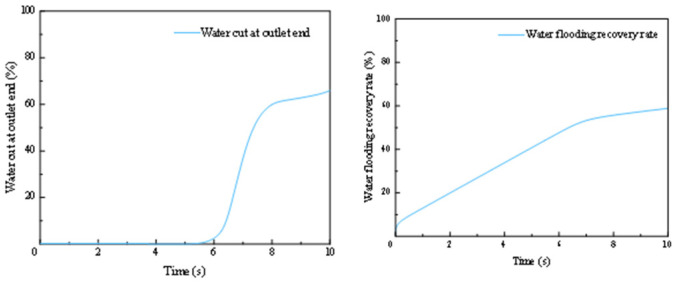
Water cut and recovery rate during water flooding.

The results of the simulation are detailed in Figure 12. The water content at the outlet is 0 for the first 5 s of the simulation. Between 5 and 8 s, it gradually increases to 60%. From 8 to 10 s, the water content continues to rise at a slower rate, reaching 66% at the 10 s mark. The recovery rate shows a rapid initial increase to 5.6% within the first 0.1 s. It then rises slowly to 54% by the 7 s mark. The rate of increase becomes more gradual after that, reaching a final value of 59% at 10 s.

#### 2.3.3. Weak Gel Foam Flooding

**Figure 13:**
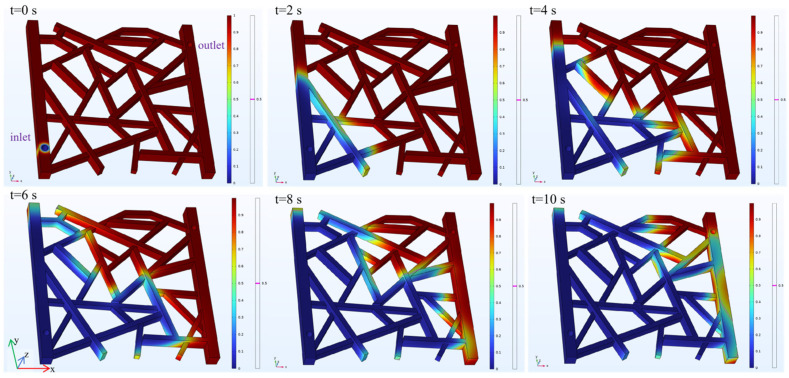
Fluid distribution during the foam flooding process.

The simulation of the foam flooding process models the distribution of the foam fluid and the oil phase. In the visualizations, red represents the simulated oil, and blue represents the foam fluid, although the color transition in the legend is more complex. A magenta line indicates the 50% volume fraction interface between the two fluids. A boundary probe is added at the model outlet to record changes in the foam content, while a domain probe is applied to the entire model to track the oil recovery rate. As shown in Figure 13, snapshots of the fluid distribution are taken every 2 s to observe the dynamic changes over the 10 s period.

**Figure 14:**
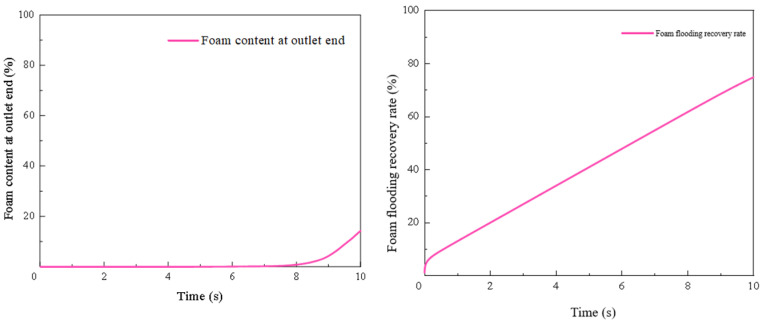
Foam content and recovery rate during foam flooding.

The results of the simulation are detailed in Figure 14. The foam content at the outlet remains at 0 for the first 7 s, indicating that no foam fluid had exited the model. From 7 to 10 s, the foam content gradually increases, rising to 14%. The recovery rate shows a rapid initial increase to 6% within the first 0.1 s. Afterward, the recovery rate increases slowly and steadily, reaching 75% by the 10 s mark.

When comparing the three displacement methods, the oil recovery rate for foam flooding shows a continuous and steady increase. The foam content at the outlet is significantly lower than the gas or water content in the other two methods, which indicates that the foam has a stronger mobility control ability and delays the breakthrough of the displacing phase. The final recovery rate reaches 75%. It is also evident from the residual oil distribution maps that foam flooding effectively expands the sweep efficiency, leading to a higher recovery rate.

In all three flooding simulations (gas, water, and foam), the geometry and boundary conditions were identical; only fluid properties (density/viscosity and mobility) were changed among cases. Gravity was included along the z-direction, leading to clear vertical segregation: gas flooding shows override (gas tends to the upper region) and water flooding shows underride (water tends to the lower region), both reducing sweep efficiency. In contrast, foam provides stronger mobility control (higher apparent viscosity/resistance), which suppresses segregation and channeling, delays breakthrough, and promotes a more uniform displacement front, resulting in higher recovery than gas and water under the same setup.

## 3. Conclusions

This study successfully formulated and evaluated a polymer-enhanced foam system for enhanced oil recovery in fractured reservoirs. The key findings are summarized as follows:

(1) The optimal formulation—0.5% SDS combined with 1000 mg·L^−1^ polymer—was determined through systematic screening of seven foaming agents. The polymer formed a 3D weak gel network in the aqueous phase, which effectively stabilized foam lamellae and improved elasticity and mechanical strength. This structure significantly extended foam lifespan and enhanced the overall system stability.

(2) At 0.7 PV, polymer foam forms a dense bank and advances piston-like propulsion, boosting areal/vertical sweep and delaying breakthrough. At 0.3 PV, effects remain localized; at 0.1 PV, gel foam rapidly plugs dominant channels, producing high residual resistance but limited front propagation. Under gravity segregation, foam fills upper gas-override zones, suppressing up-coning and deferring gas breakthrough. A practical strategy is “plug first, then push”: gel foam for conformance, polymer foam for sweep.

(3) Numerical simulations of oil displacement in a 3D fractured network model confirmed the superiority of the enhanced foam system. Foam flooding achieved a final oil recovery rate of 75%, which was considerably higher than the recovery rates achieved by gas flooding (65%) and water flooding (59%). The delayed breakthrough and improved vertical sweep confirmed the gel’s central role in controlling flow, with foam acting as an adaptive reinforcement.

(4) The polymer–gel–foam system establishes a synergistic technology chain: polymers enable viscosity control, gels provide 3D network conformance, and foams enhance sweep efficiency. As the primary material, gels inherit polymer viscosity while strengthening stability via cross-linking; as an auxiliary phase, foams further boost displacement efficiency under gel-established mobility control.

This formulation demonstrates that polymer-based weak gels can serve as the principal EOR material, with foam and nanoparticles providing complementary reinforcement under complex reservoir conditions. Future work should focus on optimizing the gel structure property relationships and scaling performance under thermal and chemical stresses typical of field deployment.

This study used a model oil; however, real crude oils may contain asphaltenes and other interfacially active components that can adsorb at gas–liquid and oil–water interfaces, alter interfacial properties/emulsification, and thereby affect foam lamella stability and mobility control. Future work should evaluate gel–foam formulations with crude oils of different asphaltene contents, combined with interfacial measurements and displacement tests, to quantify asphaltene impacts on foam performance.

## 4. Materials and Methods

### 4.1. Formulation and Performance Evaluation of Polymer Gel Foam

#### 4.1.1. Material

Foaming agents A, B, and YF-1 are provided by Shengli Oilfield (Dongying, China). Sodium dodecyl sulfate (SDS) with a purity of 97% is purchased from Shanghai Macklin Biochemical Co., Ltd. (Shanghai, China). Foaming agents ZK-1, ZK-2, and ZK-3 are supplied by Qingtian Zhongke Plant Technology Co., Ltd. (Lishui, China). Each has a 7% concentration and a molecular weight of 8 kDa. NaCl with a purity of 99.5% is purchased from Shanghai Macklin Biochemical Co., Ltd. Deionized water is prepared using an ultrapure water system to a resistivity of 18.25 MΩ·cm. SDS + polymer is used for foaming/stability evaluation and to define the optimal condition.

#### 4.1.2. Apparatus

**Figure 15:**
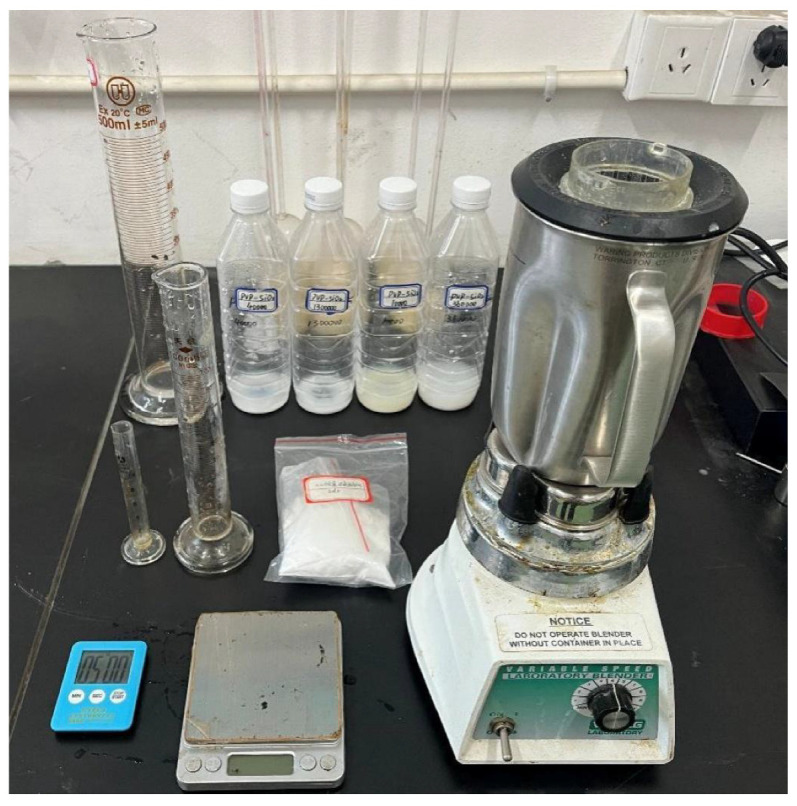
Experimental apparatus.

The following equipment is used for the experiments, as depicted in Figure 15. It consists of a Waring blender (waring commercial, Torrington, CT, USA), a timer, graduated cylinders (10 mL, 100 mL, and 1000 mL), and an electronic balance.

#### 4.1.3. Experimental Procedure

(1) Basic Performance Evaluation

The objective of this procedure is to evaluate the foaming performance of different foaming agents and determine their optimal concentrations. Solutions of seven different foaming agents are prepared in 100 mL volumes with deionized water. The final concentrations for each agent are 0.1%, 0.3%, 0.5%, 0.7%, and 1%. Each 100 mL solution is poured into a Waring blender and agitated at 8000 rpm for 3 min. The generated foam is immediately transferred to a 1000 mL graduated cylinder. The total foam volume is recorded. The time required for 50 mL of liquid to drain out is then measured and recorded as the drainage half-life. After each experiment, the blender and graduated cylinder are thoroughly rinsed with deionized water to remove any foam residue. Each test is repeated three times, and the average value is used to ensure accuracy.

(2) Polymer Synergy Evaluation

This experiment is designed to investigate the effect of a polymer on the foaming performance of SDS. A 1000 mg·L^−1^ polymer mother liquor is prepared. Then, 0.1 g of polymer is slowly added to 99.4 g of deionized water, stirring at 400 rpm. The mixture is then stirred for 1 h at 600 rpm. A 100 mL test solution is created by adding 0.5 mL of SDS to the polymer mother liquor. This final solution is agitated in the Waring blender at 8000 rpm for 3 min. The subsequent steps for measuring the foam volume and drainage half-life are identical to those described in the basic performance evaluation.

### 4.2. Weak Gel Foam Flow Experiment

#### 4.2.1. Material

The simulated crude oil used in this experiment is prepared from kerosene, paraffin oil, and oil-red dye, and the aqueous phase is stained with deionized water and methyl blue. The foaming agent used is YF-1, provided by Shengli Oilfield, and the polymer is QC-9 to enhance foam stability. The cross-linking agents of the frozen gel foam system are hydroquinone and ulotropine to improve the sealing ability of the foam in the hyperosmotic channel. YF-1 and QC-9 are commercial codes corresponding to the foaming agent and polymer used in the visual experiments; their mapping and usage scenarios will be stated explicitly. Hydroquinone + urotropine are only used for reinforced plugging (foamed gel/frozen gel foam).

#### 4.2.2. Apparatus

**Figure 16:**
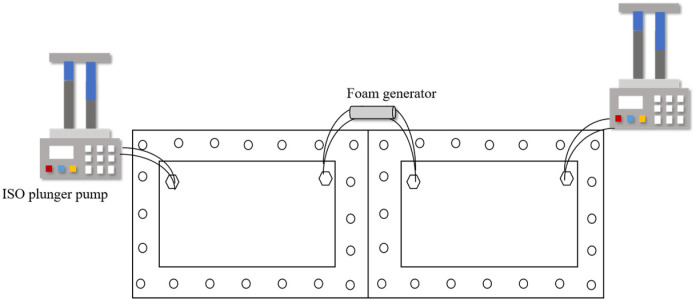
Experimental process.

As shown in Figure 16, the foam flow experimental device used in this study includes a transparent two-dimensional visualization model (dual cavity), two constant flow ISO plunger pumps (2 mL·min^−1^), a foam generator, and pipelines. The transparent dual-cavity 2D visual model is an intentionally designed heterogeneous system consisting of a dominant high-permeability channel (preferential flow path) and an adjacent low-permeability zone. All horizontal-model tests were conducted in the same dual-cavity 2D visual model with identical geometry and unchanged interstitial spacing; the inlet/outlet port locations were fixed for all runs (inlet for fluid injection and outlet for effluent production), and only the injected PV was varied among Figure 5, Figure 6 and Figure 7.

### 4.3. Model Setup and Scheme Design for Oil Displacement Simulation

**Figure 17:**
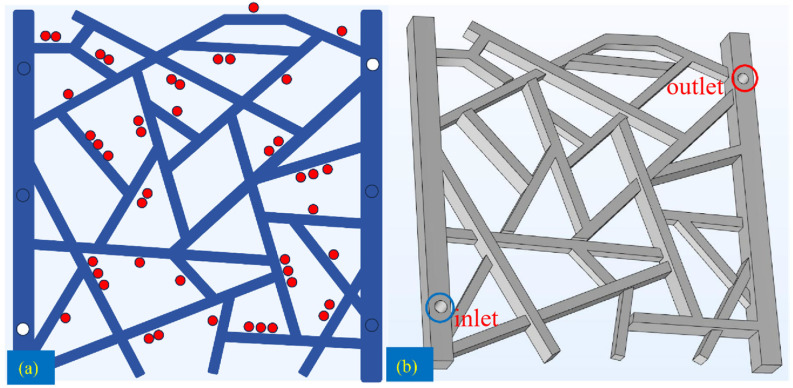
Structure of the fractured network model: (**a**): a 2D top view of the model; (**b**): 3D structure.

The structure of the fractured network model is shown in Figure 17. Figure 17a presents a 2D top view of the model, which is a 1:1 scale representation of the physical model used in the experiments. In this view, white circles indicate the inlet and outlet ports. Red circles are used to represent the depth of adjacent fractures: one circle signifies a depth of 50 µm, two circles indicate 75 µm, and three circles represent a depth of 100 µm. These fracture depths (50/75/100 µm) were specified during the design and fabrication of the physical visual model (and verified after fabrication when applicable) and were directly adopted as geometric inputs for constructing the 3D simulation domain; therefore, they were not arbitrarily assumed or fitted parameters. Figure 17b displays the 3D structure, which incorporates these specified depths. For the simulation, the fluid inlet is marked by a blue circle, and the model outlet is marked by a red circle.

**Figure 18:**
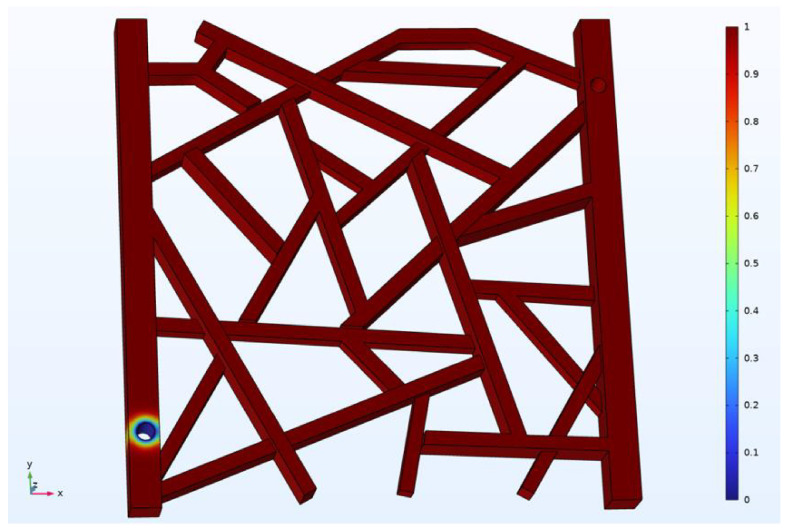
Illustration of the model saturated with oil.

As illustrated in Figure 18, the model is initially fully saturated with a simulated, red-colored oil. The oil has a viscosity of 100 mPa·s. The simulation is run for a total of 10 s with a calculation time step of 0.001 s. The fluid velocity at the inlet is set to 0.01 m·s^−1^. To observe the oil recovery effects of different technologies, three displacement scenarios are simulated: gas flooding (using nitrogen), water flooding, and foam flooding.

## Data Availability

The data that support the findings of this study are available from the corresponding author upon reasonable request.
